# Serum Total Bile Acids in Relation to Gastrointestinal Cancer Risk: A Retrospective Study

**DOI:** 10.3389/fonc.2022.859716

**Published:** 2022-06-08

**Authors:** Songbo Li, Xiaodong Qu, Luyao Zhang, Na Wang, Min Chen, Xingyu Zhao, Jie Wang, Huanhuan Lv, Ying Qi, Lifeng Zhang, Junye Liu, Yongquan Shi

**Affiliations:** ^1^ State Key Laboratory of Cancer Biology, National Clinical Research Center for Digestive Diseases, Xijing Hospital, Air Force Medical University, Xi’an, China; ^2^ School of Clinical Medicine, Xi’an Medical University, Xi’an, China; ^3^ Department of Radiation Protective Medicine, School of Military Preventive Medicine, Air Force Medical University, Xi’an, China

**Keywords:** gastrointestinal cancer (GI cancer), serum, bile acid (BA), biomarkers, epidemiology - analytic (risk factors)

## Abstract

**Background:**

Bile acids (BAs) have been proposed to promote gastrointestinal cells carcinogenesis. However, studies on serum total bile acid (TBA) levels and gastrointestinal cancers (GICs) risk are rare.

**Methods:**

We conducted a retrospective case–control study from 2015 to 2019 at the First Affiliated Hospital of Air Force Military Medical University, in which 4,256 GICs cases and 1,333 controls were recruited. Patients’ demographic, clinical and laboratory data were collected. The odds ratios (ORs) with 95% confidence intervals (CIs) were estimated using binary logistic regression models.

**Results:**

Positive associations were observed between serum TBA levels and risks of esophageal cancer (EC), gastric cancer (GC) and colorectal cancer (CRC). Overall, ORs of EC, GC and CRC risk rose with the TBA levels increasing. After adjustment for potential confounders, the OR of TBA-positive for EC risk was 4.89 (95% CI: 3.20-7.49), followed by GC (OR: 3.92, 95% CI: 2.53-6.08), and CRC (OR: 3.32, 95% CI: 2.04-5.11). Patients aged 60 years or older have a higher risk of GICs, especially for EC patients. Males are associated with a higher risk of GC, while females are associated with a higher risk of CRC. Preoperative serum TBA positive and negative was significantly different in the presence or absence of hematogenous metastasis among EC patients (*P*=0.014), and lymph node metastasis among GC patients (*P*=0.018).

**Conclusions:**

This retrospective study showed positive associations between serum TBA level and GICs risk, and a higher serum TBA level constitutes a risk factor for GICs.

## Introduction

Global cancer incidence and mortality are rising rapidly, with nearly 19.3 million new cases and 10.0 million deaths worldwide in 2020 (1). Approximately 14.7% of these new cases are gastrointestinal cancers (GICs, including esophageal, gastric and colorectal cancers), and both their morbidity and mortality are high in East Asia (1). In China, esophageal cancer (EC), gastric cancer (GC) and colorectal cancer (CRC) remain the top five most common and deadly tumors (2), and the total numbers of new cases and deaths caused by GICs will continue to increase due to changes in the population’s age and structure (3). GICs are multifactorial and complex pathological processes that are influenced by carcinogenic factors such as diet, lifestyle, environmental factors and genetic susceptibility. Hence, understanding the potential risk factors of GICs is essential for cancer prevention efforts. Recently, some studies have reported a link between bile acids (BAs) and GICs (4–7).

BAs is a normal component of intestinal contents, which is released into the intestine during digestive activities to promote the digestion and absorption of lipids and fat-soluble vitamins (8). Apart from functioning to promote lipid absorption as a detergent, BAs also act as endogenous ligands to regulate metabolic pathways by activating several nuclear and plasma membrane receptors (9). These receptors are extensively expressed in a variety of human tissues, including liver, bile duct, stomach, intestine and other digestive organs, with the nuclear receptor farnesoid X receptor (FXR) and the cell membrane surface G protein-coupled receptor TGR5 (G protein-coupled bile acid receptor 1) being the most studied (10). Although BAs exists as a normal physiological component in the body, it was proposed as a carcinogen as early as the 1940s (11). BAs can directly damage cells, including the induction of DNA damage, mutations and a reduction in apoptotic capacity (12), and recently there is growing evidence that chronic exposure of gastrointestinal cells to high physiological levels of BAs may induce carcinogenesis both *in vitro* and *in vivo* studies (13, 14). In addition, the high levels of BAs in the gastrointestinal tract of patients with gastric and colorectal cancers have also been confirmed (15, 16). However, whether serum total TBA levels are correlated with GICs has been reported less frequently. Therefore, we designed this retrospective case-control study to identify whether TBA levels are an associated risk factor for GICs.

## Material and Methods

### Study Design and Participants

We retrospectively reviewed the medical records of 12,566 patients who received inpatient care at the First Affiliated Hospital of Air Force Military Medical University from January 2015 to December 2019. We included all patients with surgically and histopathologically confirmed gastrointestinal cancers (including EC, GC and CRC) as cases and included healthy check-up patients at the health medicine center as controls. All the patients were aged 18-75 years and of any gender. The exclusion criteria were: 1) previous diagnosis of GICs with radiotherapy or chemotherapy prior to surgery; 2) history of digestive system surgery (including esophagus, stomach, intestine and liver); 3) malignancy with other systems; 4) diagnosis of inflammatory bowel disease; 5) autoimmune diseases such as primary biliary cirrhosis, primary sclerosing cholangitis, IgG4-related sclerosing cholangitis, and autoimmune liver disease; 6) chronic liver diseases (including viral hepatitis, alcoholic hepatitis, non-alcoholic steatohepatitis, and drug-related hepatitis); 7) incomplete data and information. Finally, a total of 5589 cases (4256 cases of GIC and 1333 cases of control) were eligible for inclusion and were therefore included in the study.

This study was approved by the Ethics Committee of the First Affiliated Hospital of Air Force Military Medical University and was undertaken in accordance with the Declaration of Helsinki.

### Data Collection

**Figure 1:**
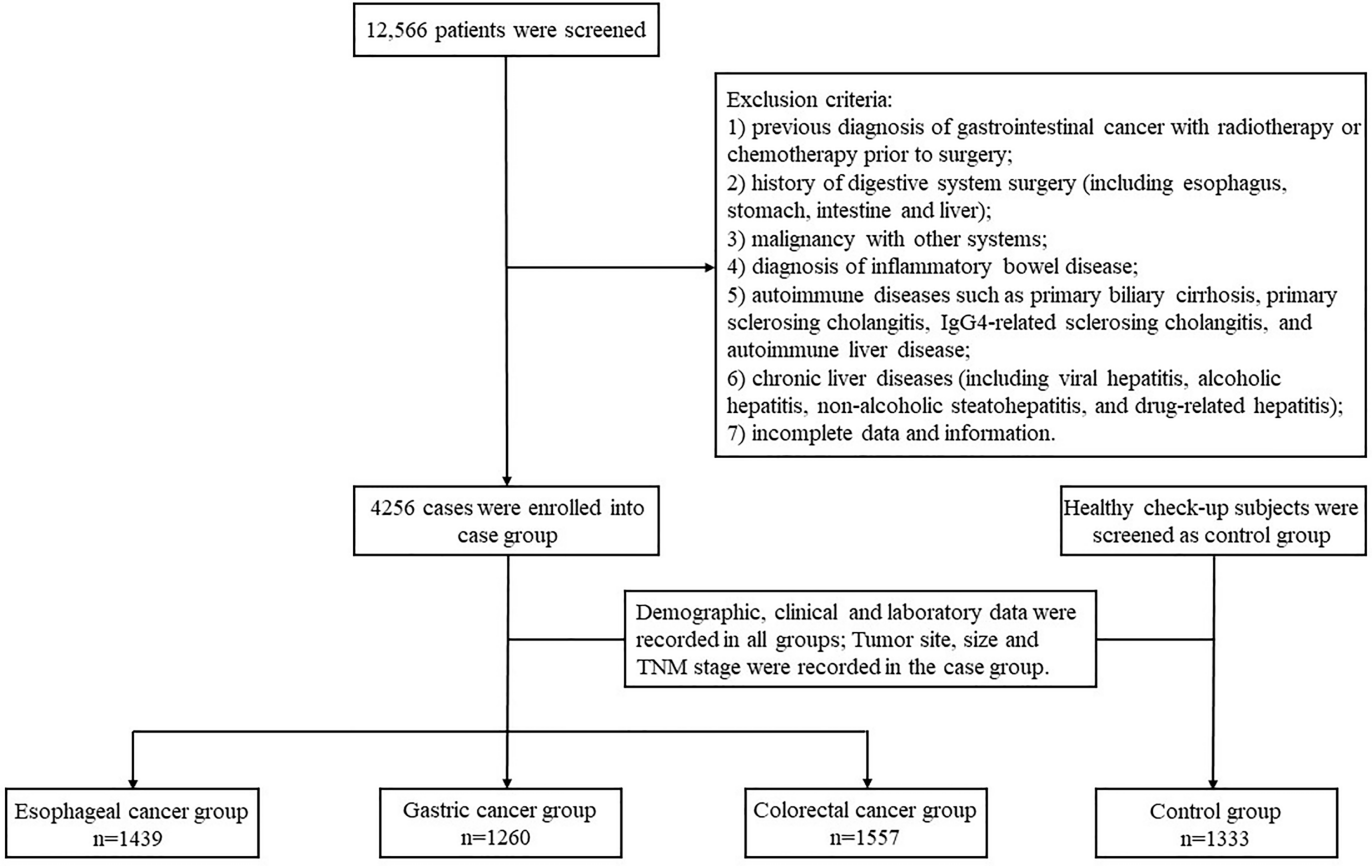
Flow chart of participant enrollment.

Demographic, clinical and laboratory data were collected from patients. Demographic data included gender, age, marital status, and residence. Laboratory data included serum TBA, CEA, AFP, CA19–9 and CA125 levels and were detected from the day before operation. The cut off value of TBA, CEA, AFP, CA19–9 and CA125 levels were 10μmol/L, 5 ng/ml, 7 ng/ml, 30 U/ml, 24 U/ml. Tumor site, size and lymph node metastasis were defined by pathologists in the department of pathology according to the Tumor-Node-Metastasis (TNM)-based staging of esophageal, gastric, and colorectal cancer by the new, 8th editions of the relevant Union for International Cancer Control (UICC) and American Joint Committee on Cancer (AJCC) publications. The flow chart of the study design is shown in **Figure 1**.

### Statistical Analysis

Data collected were encoded into numbers and statistically analyzed. The mean ± standard deviation (x̅ ± s) was used to describe characteristics that conform to a normal distribution, and the median and interquartile spacing are used for those that do not. For count data, the frequency in percentage (%) was presented. The chi-square (χ2) test and Fisher’s exact test were used to examine the discrete variables. The non-parametric Mann-Whitney U test was used to analyze whether there were differences in levels of serum TBA and tumor makers between the case and control groups. The restricted cubic spline regression models with five knots were performed to estimate the nonlinear relationship of serum TBA levels with the risk of esophageal, gastric, colorectal and gastrointestinal cancer. We applied binary logistic regression models to analyze the relationship between serum TBA levels and GICs risk, and the ORs with 95% CIs were calculated. All analyses were performed using STATA version 15.0 (Stata Corporation) and SPSS version 25.0 (IBM, SPSS Inc., Chicago, IL, US). Two‐sided P values less than 0.05 were considered statistically significant.

## Results

### Demographic Characteristics of the Study Participants

The case group included 4,256 participants. Of these, 1,439 (33.8%) were EC, 1,260 (29.6%) were GC, and 1,557 (36.6%) were CRC. The control group included 1,333 participants. The mean age of study participants in the case group was older than that in the control group, and the proportion of males in the case group was higher than that of female patients (**Table 1**). The proportion of EC (88.5% vs. 11.5%) and GC (70.4% vs. 29.6%) patients was significantly higher in rural areas than in urban areas. Compared to the control group, GIC patients had a higher proportion of users of aspirin and metformin, and the difference was statistically significant (p < 0.05; **Table 1**). However, due to the small number of users due to aspirin, metformin and statins in both the case group and the control group, the relevant data in this study may not truly reflect the relationship between the drugs and GIC. The other detailed demographic characteristics are shown in **Table 1**.

**Table 1:** Characteristics of the study participants in the case group and the control group, numbers (%).

Characteristics	Case groups				Control group (n=1333)
	EC (n=1439)	GC (n=1260)	CRC (n=1557)	GICs (total) (n=4256)	
Age, y (mean ± SD)	62.31 ± 7.27^a^	57.27 ± 9.40^a^	58.31 ± 10.26 ^a^	59.35 ± 9.33^a^	55.40 ± 9.52
Sex					
Male Female	976 (67.8%) 463 (32.2%)	959 (76.1%)^a^ 301 (23.9%)	911 (58.5%)^a^ 646 (41.5%)	2846 (66.9%) 1410(33.1%)	889 (66.7%) 444 (33.3%)
BMI, kg/m² (mean ± SD)	23.08 ± 3.50^a^	22.78 ± 3.18^a^	23.60 ± 3.47	23.24 ± 3.28^a^	24.01 ± 3.14
Marital status					
Married Unmarried/divorced	1390 (96.6%)^a^ 49 (3.4%)	1254 (99.5%) 6 (0.5%)	1551 (99.6%) 6 (0.4%)	4195 (98.6%)^a^ 61 (1.4%)	1326 (99.5%) 7 (0.5%)
Residence					
Urban Rural	165 (11.5%)^a^ 1274 (88.5%)	373 (29.6%)^a^ 887 (70.4%)	891 (57.2%)^a^ 666 (42.8%)	1429 (33.6%)^a^ 2827 (66.4%)	1007 (75.5%) 326(24.5%)
History of biliary tract disease^b^					
Yes No	52 (3.6)^a^ 1387 (96.4%)	103(8.2%) 1157 (91.9%)	83(5.3%)^a^ 1474 (94.7%)	238 (5.6%)^a^ 4018 (94.4%)	127 (9.5%) 1206 (90.5%)
History of cholecystectomy					
Yes No	27 (1.9%)^a^ 1412(98.1%)	109(8.7%) 1151 (91.3%)	75 (4.8%)^a^ 1482 (95.2%)	211(5.0%) 4045 (95.0%)	117 (8.8%) 1216 (91.2%)
History of hypertension					
Yes No	308 (21.4%) 1131 (78.6%)	191 (15.2%)^a^ 1069 (84.8%)	253 (16.2%)^a^ 1304 (83.8%)	752 (17.7%) 3504 (82.3%)	264 (19.8%) 1069 (80.2%)
History of coronary heart disease					
Yes No	98 (6.8%)^a^ 1341 (93.2%)	44 (3.5%) 1216 (96.5%)	40 (2.6%) 1517 (97.4%)	182 (4.3%) 4074 (95.7%)	45 (3.4%) 1288 (96.6%)
History of diabetes					
Yes No	104 (7.2%)^a^ 1335 (92.8%)	81 (6.4%)^a^ 1179 (93.6%)	103 (6.6%)^a^ 1454 (93.4%)	288 (6.8%)^a^ 3968 (93.2%)	62 (4.7%) 1271 (95.3%)
Use of aspirin					
Yes No	58 (4.0%)^a^ 1381 (96.0%)	49 (3.9%)^a^ 1211 (96.1%)	47 (3.0%)^a^ 1510 (97.0%)	154 (3.6%)^a^ 4102(96.4%)	23 (1.7%) 1310 (98.3%)
Use of metformin					
Yes No	42 (2.9%)^a^ 1397 (97.1%)	39 (3.1%)^a^ 1221 (96.9%)	56 (3.6%)^a^ 1501 (96.4%)	137 (3.2%)^a^ 4119 (96.8%)	23 (1.7%) 1310 (98.3%)
Use of statin					
Yes No	40 (2.8%)^a^ 1399 (97.2%)	16 (1.3%) 1244 (98.7%)	15 (1.0%) 1542 (99.0%)	71 (1.7%) 4185 (98.3%)	20 (1.5%) 1313 (98.5%)

a P value < 0.05 compared with the control group.

b Biliary tract diseases include cholecystitis, gallstones, and gallbladder polyps.

SD, standard deviation; EC, esophageal cancer; GC, gastric cancer; CRC, colorectal cancer; GICs, gastrointestinal cancers.

### Comparison of TBA and Tumor Markers Between Case and Control Groups

Preoperative tumor markers and TBA levels were compared between cases and control group. Median TBA level in EC patients (3.90μmol/L) tended to be higher than that in the control group (2.75μmol/L), and the difference was statistically significant (p < 0.05; **Table 2**). Median CEA levels were higher among three case groups than that in the control group, particularly for CRC patients, and the difference was all statistically significant (p < 0.05; **Table 2**). Whereas, median AFP levels between the case groups and the control group showed no statistical difference (**Table 2**). In the GC group and CRC group, the median levels of CA19-9 and CA125 were higher than that in the control group, and showed similar median values and distributions (**Table 2**). In addition, to identify the specificity and sensitivity of these serum markers to diagnosis, we calculated their Area Under Curve (AUC). The AUC value of CEA among CRC patients was the highest, at 0.721. Whereas only the AUC value of TBA among EC patients was statistically significant in case group, at 0.640. Relevant information is shown in **Supplementary Table S1**.

**Table 2:** TBA and tumor markers among patients and control individuals.

Factors	Case median (IQR)				Control median (IQR) (n=1333)
	EC (n=1439)	GC (n=1260)	CRC (n=1557)	GICs (total) (n=4256)	
TBA (μmol/L)	3.90 (4.27)^a^	2.80 (3.47)	2.60 (2.84)	3.10 (3.63)^a^	2.75 (2.86)
CEA (ng/ml)	1.99 (1.73)^a^	2.25 (2.51)^a^	3.27 (5.29)^a^	2.40 (2.77)^a^	1.76 (1.51)
AFP (ng/ml)	2.77 (2.16)	2.73 (1.94)	2.75 (1.72)	2.75 (1.91)	2.79 (1.78)
CA199 (U/ml)	9.27 (8.68)	9.80 (13.08)^a^	12.40 (15.08)^a^	10.44 (11.84)^a^	9.13 (8.54)
CA125 (U/ml)	8.60 (5.94)	10.64 (6.88) ^a^	11.06 (7.78)^a^	10.14 (6.96)^a^	8.66 (5.76)

a P value < 0.05 compared with the control group.

IQR, interquartile range; TBA, total bile acid; EC, esophageal cancer; GC, gastric cancer; CRC, colorectal cancer; GICs, gastrointestinal cancers.

### TBA and Gastrointestinal Cancers Risk

**Figure 2:**
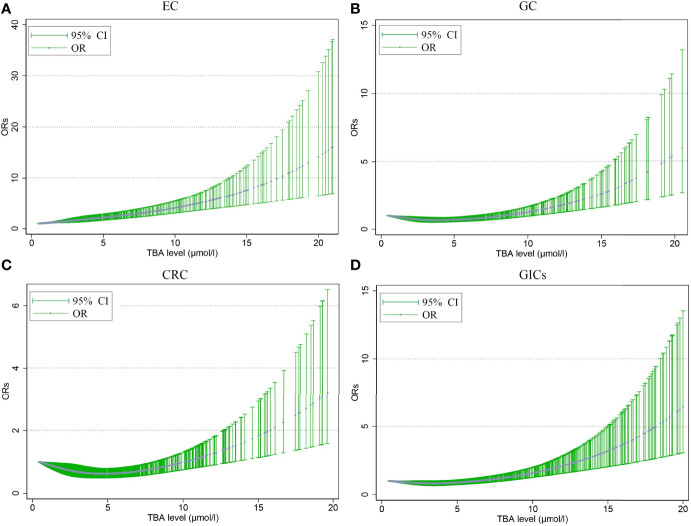
The nonlinear association between different serum TBA levels adjusted for age and the risk of EC **(A)**, GC **(B)**, CRC **(C)**, GIC **(D)**. The trend fitted by the blue dots presents the OR values estimated by the restricted cubic spline regression model. The dot indicates that there is at least one actual value of the X axis. TBA, total bile acid; EC, esophageal cancer; GC, gastric cancer; CRC, colorectal cancer; GIC, gastrointestinal cancer; OR, odds ratio; CI, confidence interval.

**Figure 2** shows the non-linear relationship between different TBA levels adjusted for age and the risk of GIC. The ORs of EC rose with the TBA levels increasing, and the ORs of GC nearly leveled off before the TBA level reached 10 μmol/L. After that, the ORs of EC and GC rose rapidly, and reached around 15.0 and 5.0 at 20 μmol/L, respectively (**Figures 2A, B**). Before reaching 10 μmol/L, TBA levels showed a U-shape relation with CRC risk, with the nadir at 5 μmol/L; after that, the ORs sharply rose to around 3.0 at 20 μmol/L (**Figure 2C**).

**Figure 3:**
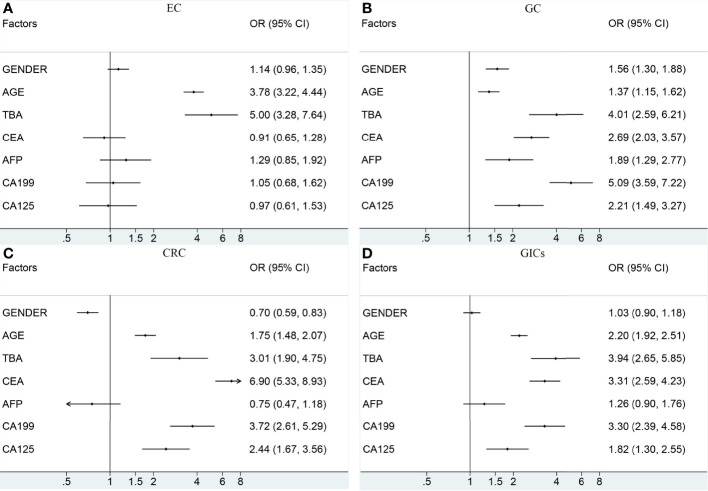
The association between the potential risk factors and the risk of EC **(A)**, GC **(B)**, CRC **(C)**, GIC **(D)**. The black dot indicates the OR of each category. The horizontal line represents the 95% CI. TBA, total bile acid; EC, esophageal cancer; GC, gastric cancer; CRC, colorectal cancer; GIC, gastrointestinal cancer; OR, odds ratio; CI, confidence interval.

To further understand the relationship between the potential risk factors with GICs, binary logistic regression analysis was also performed (**Figure 3** and **Table 3**). Patients were divided into TBA-positive and TBA-negative by cut-off value of TBA of 10μmol/L. We observed statistically a significant positive association between serum TBA levels and GICs risk when comparing the control group (**Figure 3** and **Table 3**). The strongest association was between TBA-positive and EC risk (OR: 5.00, 95% CI: 3.28 to 7.64; P <0.001), followed by GC (OR: 4.01, 95% CI: 2.59 to 6.21; P <0.001), and CRC (OR: 3.01, 95% CI: 1.90 to 4.75; P <0.001). We further analyzed the association of patients’ demographic, and laboratory data with the risk of GIC. Although various exposure factors, such as history of drugs and cholecystectomy, were statistically different in the demographics of the study participants, we included only a subset of them due to the robustness of the data. The results also indicate that patients aged 60 years or older have a higher risk of GICs, especially for EC patients (OR: 3.78, 95% CI: 3.22 to 4.44; P <0.001). Males are linked with a higher risk of GC, while females are associated with a higher risk of CRC (**Figure 3** and **Table 3**). The CEA, CA19-9, and CA125 were positively correlated with the risk of GC and CRC (**Figure 3** and **Table 3**). CEA showed a strong association with CRC risk (OR: 6.90, 95% CI: 5.33 to 8.93; P <0.001), and CA19-9 showed a strong association with GC risk (OR: 5.09, 95% CI: 3.59 to 7.22; P <0.001).

**Table 3:** ORs and 95% CIs for GICs in association with patients’ demographic, clinical and laboratory data by binary logistic regression (N=5589)^a^.

	EC (n=1439)		GC (n=1260)		CRC (n=1557)		GICs (n=4256)	
	OR (95% CI)	*P*	OR (95% CI)	*P*	OR (95% CI)	*P*	OR (95% CI)	*P*
Gender								
Female Male	1.00 1.14 (0.96,1.35)	0.141	1.00 1.56 (1.30, 1.88)	<0.001	1.00 0.70 (0.59, 0.83)	<0.001	1.00 1.03 (0.90, 1.18)	0.701
Age								
≤60 > 60	1.00 3.78 (3.22, 4.44)	<0.001	1.00 1.37 (1.15, 1.62)	<0.001	1.00 1.75 (1.48, 2.07)	<0.001	1.00 2.20 (1.92, 2.51)	<0.001
TBA (μmol/L)								
≤10 > 10	1.00 5.00 (3.28, 7.64)	<0.001	1.00 4.01 (2.59, 6.21)	<0.001	1.00 3.01 (1.90, 4.75)	<0.001	1.00 3.94 (2.65, 5.85)	<0.001
CEA (ng/ml)								
≤5.0 > 5.0	1.00 0.91 (0.65,1.28)	0.597	1.00 2.69 (2.03, 3.57)	<0.001	1.00 6.90 (5.33, 8.93)	<0.001	1.00 3.31 (2.59, 4.23)	<0.001
AFP (ng/ml)								
≤7.0 > 7.0	1.00 1.29 (0.85, 1.92)	0.237	1.00 1.89 (1.29, 2.77)	0.001	1.00 0.75 (0.47, 1.18)	0.209	1.00 1.26 (0.90, 1.76)	0.172
CA19-9 (U/ml)								
≤30.0 > 30.0	1.00 1.05 (0.68, 1.62)	0.812	1.00 5.09 (3.59, 7.22)	<0.001	1.00 3.72 (2.61, 5.29)	<0.001	1.00 3.30 (2.39, 4.58)	<0.001
CA125 (U/ml)								
≤24.0 > 24.0	1.00 0.97 (0.61, 1.53)	0.881	1.00 2.21 (1.49, 3.27)	<0.001	1.00 2.44 (1.67, 3.56)	<0.001	1.00 1.82 (1.30, 2.55)	0.001

a The control group for all comparisons is non-gastrointestinal cancer individuals.

TBA, total bile acid; EC, esophageal cancer; GC, gastric cancer; CRC, colorectal cancer; GICs, gastrointestinal cancers; OR, odds ratio; CI, confidence interval.

### Biomarkers and Clinicopathological Parameters of Gastrointestinal Cancer

To determine whether serum TBA levels are linked with GICs pathological parameters, we further evaluated corrections between GIC pathological parameters (i.e., lymph node metastasis, hematogenous metastasis, etc.) with TBA (**Table 4**). Chi-square test results showed that there were significant differences in serum TBA levels in EC patients in terms of age and hematogenous metastasis (p < 0.05; **Table 4**). The serum TBA levels were also significantly different in gender, age and the presence or absence of lymph node metastasis among GC patients (p < 0.05; **Table 4**). In contrast, there was no significant difference between serum TBA levels and clinicopathological parameters in CRC patients. What’s more, no significant difference was observed between serum TBA levels and clinical tumor-node-metastasis (mTNM) stage (P>0.05; **Table 4**).

**Table 4:** Comparison of clinicopathological characteristics between two groups stratified by TBA level in the case group.

	EC=1439				GC=1260				CRC=1557				GICs (total)			
Characteristics	TBA (+)	TBA (-)	*P*	χ2	TBA (+)	TBA (-)	*P*	χ2	TBA (+)	TBA (-)	*P*	χ2	TBA (+)	TBA (-)	*P*	χ2
Gender																
Male Female	104 50	872 413	0.934	0.007	90 17	869 284	0.042	4.117	56 36	855 610	0.636	0.224	250 103	2596 1307	0.100	2.713
Age																
≤60 > 60	43 111	472 813	0.031	4.644	50 57	690 463	0.008	6.948	45 47	782 683	0.405	0.693	138 215	1944 1959	<0.001	14.872
Lymphatic metastasis																
Yes No	80 74	640 645	0.615	0.253	81 26	742 411	0.018	5.565	45 47	682 783	0.660	0.194	206 147	2064 1839	0.048	3.898
Hematogenous metastasis																
Yes No	8 146	26 1259	0.014	5.996	3 104	19 1134	0.383	0.763	4 88	60 1405	0.906	0.014	15 338	105 3798	0.090	2.872
cTNM^a^ stage																
Stage I Stage II Stage III Stage IV	24 89 26 15	270 642 221 152	0.244	4.168	20 21 63 3	283 302 549 19	0.078^b^	6.518	13 34 41 4	248 531 626 60	0.917^b^	0.525				

a Clinical Tumor-Node-Metastasis (TNM)-based staging of esophageal, gastric, and colorectal cancer by the new, 8th editions of the relevant Union for International Cancer Control (UICC) and American Joint Committee on Cancer (AJCC) publications.

b Fisher exact test.

TBA, total bile acid; EC, esophageal cancer; GC, gastric cancer; CRC, colorectal cancer; GICs, gastrointestinal cancers; OR, odds ratio; CI, confidence interval.

## Discussion

In this retrospective study, we investigated whether preoperative serum TBA levels were associated with the risk of GICs. We observed that levels of serum TBA were statistically significantly associated with increased GIC risk, especially after reaching 10μmol/L. In order to explore the deeper relationship between serum TBA levels and GICs, we further assessed corrections between GIC pathological parameters. Besides, we also analyzed the relationship between some potential risk factors with GICs, including age, gender, medical history and tumor markers. Overall, these findings suggest that a higher level of TBA may promote GICs.

BA plays a crucial role in human health and disease, with dissolving nutrients such as dietary lipids and fat-soluble vitamins, thereby significantly enhancing their intestinal absorption (17). Besides, BAs are also signaling molecules to produce metabolic effects by interacting with FXR and TGR5, PXR and VDR (18), which act predominantly in enterohepatic tissues, but also in peripheral organs. Once cholestasis occurs for a variety of reasons, which is characterized by elevated levels of BA in the liver and serum, followed by hepatocyte and biliary injury (19), inflammation (20), metabolic disorders and other diseases (21, 22). What’s more, BA is a potential cancer promoter, which plays a crucial role in regulating the proliferation of cancer cells from different sources (23). BA exposure has been considered a potential risk factor for GIC (24), and increased BA secretion is associated with an increased incidence of GIC (25). It has been reported that high concentrations of secondary BA can cause oxidative DNA damage, promote inflammation and activation of NF-κB, which in turn can damage the gastrointestinal epithelium and thus play an important role in GIC progression (26).

Previous epidemiological studies on BA and the risk of GICs were case-control studies of fecal bile acid contents, particularly with respect to CRC risk. In enterohepatic circulation, primary bile acids undergo biochemical transformation, such as dehydroxylation, by intestinal bacteria to produce secondary bile acids (deoxycholic acid and shicholic acid) (27). Secondary bile acids, particularly deoxycholic acid, have long been implicated as co-carcinogens in CRC, which may damage the colon epithelium and thus accelerate carcinogenesis (28–32). Hepatocytes secrete BAs to the bile canaliculi, and the gallbladder empties to the duodenum upon feeding and releases BAs to the gastrointestinal tract. BAs are then reabsorbed through the enterocytes and get to the liver for reuptake and reuse through the portal circulation. Therefore, gastric juice BA and serum BA have the same source, that is, both come from the gallbladder. Since deoxycholic acid is formed in the colon and is absorbed into the portal systemic circulation, serum deoxycholic acid concentration might be of value as biomarkers of risk of CRC (33). Two small case-control studies suggested that the positive association between the serum unconjugated secondary bile acids concentration and colorectal adenoma (32, 34). In addition, a prospective study showed that certain conjugated primary and secondary bile acid levels before diagnosis were positively associated with colon cancer risk (6).

Another important result of our study was that TBA-positive patients had a higher relative risk of EC (OR: 4.89, 95% CI: 3.20 to 7.49) and GC (OR: 3.92, 95% CI: 2.53 to 6.08) compared to CRC (OR: 3.32, 95% CI: 2.04 to 5.11). BA exposure has also been implicated in the development and progression of EC (35–37) and GC (38, 39). Esophageal inflammation caused by continuous reflux of bile acid‐containing gastric fluids is considered a major risk factor for EC. However, studies have shown that exposure to unmodified non-mutagenic bile acids can also induce EC even in the absence of gastric acid (40, 41), which further indicated that BAs may be related to EC risk. Munemoto et al. observed that stimulation with taurocholic acid (TCA) results in high levels of additional chromosomal alterations, and suggested that TCA‐mediated activation of G6PD accelerates the progression of cancer, including genetic alterations, by upregulating the pentose phosphate pathway and overexpressing NF‐κB (42).

The association between serum TBA levels and the risk of GIC by restricted cubic spline regression is shown in **Figure 2**. Overall, the relative risks of GICs rose with the TBA levels increasing. A retrospective study of 30,465 patients showed that the degree of damage to the gastric mucosa worsened with increasing intragastric BA concentrations (43). Another study in Japan showed that in *H.pylori* positive patients, the degree of gastric mucosal atrophy as well as intestinal metaplasia worsens with increasing BA concentrations, and those with high intragastric BA concentrations are more likely to develop GC (16). A European prospective study of 569 patients demonstrated that colon cancer risk was positively associated with plasma levels of conjugated BA, independent of unconjugated BA and tertiary BA (6). These studies all showed that the occurrence of GC was correlated with BA concentrations in the stomach, which is consistent with our research.

Gastric intestinal metaplasia (IM) induced by BAs is a precancerous lesion of gastric adenocarcinoma, which is linked with the expression of caudal-related homeobox 2 (CDX2) (44). Previously, our research group has found that the activation of FXR and the sequence of SHP directly induced transcription were involved in the expression of CDX2 in bile acid-induced gastric IM lesions (45). Our previous study also indicated that FXR may be associated with a series of molecular changes in gastric cells after BA treatment, in which the FXR/SNAI2/miR-1 axis played an important role in BA-induced IM progression (7). Besides, we also elucidated the role of the TGR5-ERK1/2-HNF4α axis during IM development in patients with BAs reflux, which may help to provide prospective strategies for IM treatment (46). However, there are still many uncertainties about how BAs affect the pathology of GICs, especially in relation to serum TBA.

In addition to the relationship between serum TBA and GIC risk, there were some interesting findings in the present study. In a binary logistic regression analysis that included both sex and TBA levels, females are found to be linked with a higher risk of CRC than males (**Table 3**). In a comprehensive prospective metabolomics study, serum glycochenodeoxycholate was most strongly associated with CRC risk among females (OR = 5.34) (4), which suggested that high TBA level might be a risk factor for females. The serum TBA level was significantly different in the presence or absence of hematogenous metastasis among EC patients (*P* =0.014), and lymph node metastasis among GC patients (*P* =0.018). Therefore, we speculated that TBA level combined with tumor markers could predict EC hematogenous metastasis and GC lymph node metastasis, thus guiding the treatment and prognosis of GIC. Besides, various studies have confirmed cholecystectomy (47) and drugs, such as aspirin (48), metformin (49) and statins (50), might exert an effect in the field of GICs. However, relevant data in this study are few and further studies are needed to elucidate the mechanism of action and its true significance in cancer prevention.

Our study was the first large retrospective study to examine the relationship between serum TBA levels and the risk of esophageal, gastric and colorectal cancer. Overall, it supports the hypothesis that BAs promotes the carcinogenesis of gastrointestinal cells. However, this study had several limitations. Firstly, despite the large case number, this study is a single-center retrospective study, which has the disadvantages of case selection bias and insufficient reliability, and the results need to be further verified by a multicenter prospective study. Secondly, other related information, such as smoking and drinking, which might modify BAs effect on GICs risk, was not collected. Thirdly, the results of serum TBA we collected in a single serum sample could not represent the daily variation of metabolites and serum TBA measurement is burdened by non-specific effects, which may attenuate the correlation. Moreover, we did not have the opportunity to compare BA levels in the blood with fecal levels in our study, which have been reported to play vital roles with gut microbiota in intestinal carcinogenesis (51). But our results indicated that exposure to the high level of BAs might be a risk factor for GICs.

In summary, we observed positive associations between serum TBA level and GICs risk, and a higher serum TBA level constitutes a risk factor for GICs. Considering serum TBA level can be manipulated by dietary modifications and medications, removing or minimizing exposures to related risk factors should be added into prevention and treatment strategies for reducing the GICs incidence. Further studies are needed to investigate the effects of specific interventions on BAs in blood and feces in humans.

## Data Availability Statement

The data analyzed in this study is subject to the following licenses/restrictions: The datasets generated and analyzed during the current study are available from the corresponding authors on reasonable request. Requests to access these datasets should be directed to Yongquan Shi, shiyquan@fmmu.edu.cn.

## Ethics Statement

The studies involving human participants were reviewed and approved by The Ethics Committee of the First Affiliated Hospital of Air Force Military Medical University and approval was obtained (date 18 November 2020, file number KY20202100-F-1). Written informed consent for participation was not required for this study in accordance with the national legislation and the institutional requirements.

## Author Contributions

SL, XQ, and LYZ retrieved and analyzed all the data in the research. NW, MC, and XZ helped conceptualize the research and edited the manuscript. JW, HL, YQ, and LFZ acquired the original data and developed the methodology. JL and YS designed, checked, and supervised the research process. All authors contributed to the article and approved the submitted version.

## Funding

This work was supported by grants from the National Natural Science Foundation of China (No. 82170560), an Independent Project of State Key Laboratory of Cancer Biology (No. CBSKL2019ZZ007) and Booster Plans of Xijing Hospital (No. XJZT21L07 and No. JSYXM04).

## Conflict of Interest

The authors declare that the research was conducted in the absence of any commercial or financial relationships that could be construed as a potential conflict of interest.

## Publisher’s Note

All claims expressed in this article are solely those of the authors and do not necessarily represent those of their affiliated organizations, or those of the publisher, the editors and the reviewers. Any product that may be evaluated in this article, or claim that may be made by its manufacturer, is not guaranteed or endorsed by the publisher.

## Abbreviations

AUC, Area Under Curve; BA, bile acid; CI, confidence interval; CRC, colorectal cancer; EC, esophageal cancer; GC, gastric cancer; GIC, gastrointestinal cancer; IM, intestinal metaplasia; OR, odds ratio; TBA, total bile acid; TCA, taurocholic acid.

## References

[1] Sung H Ferlay J Siegel RL Laversanne M Soerjomataram I Jemal A. Global Cancer Statistics 2020: GLOBOCAN Estimates of Incidence and Mortality Worldwide for 36 Cancers in 185 Countries. CA Cancer J Clin (2021) 71:209–49. doi: 10.3322/caac.21660 33538338

[2] Zheng RS Sun KX Zhang SW Zeng HM Zou XN Chen R. Report of Cancer Epidemiology in China, 2015. Zhonghua Zhong Liu Za Zhi (2019) 41:19–28. doi: 10.3760/cma.j.issn.0253-3766.2019.01.005 30678413

[3] Li S Chen H Man J Zhang T Yin X He Q. Changing Trends in the Disease Burden of Esophageal Cancer in China From 1990 to 2017 and its Predicted Level in 25 Years. Cancer Med (2021) 10:1889–99. doi: 10.1002/cam4.3775 PMC7940228 33586344

[4] Cross AJ Moore SC Boca S Huang W-Y Xiong X Stolzenberg-Solomon R. A Prospective Study of Serum Metabolites and Colorectal Cancer Risk. Cancer (2014) 120:3049–57. doi: 10.1002/cncr.28799 PMC5819589 24894841

[5] Ding L Yang L Wang Z Huang W. Bile Acid Nuclear Receptor FXR and Digestive System Diseases. Acta Pharm Sin B (2015) 5:135–44. doi: 10.1016/j.apsb.2015.01.004 PMC4629217 26579439

[6] Kühn T Stepien M López-Nogueroles M Damms-Machado A Sookthai D Johnson T. Prediagnostic Plasma Bile Acid Levels and Colon Cancer Risk: A Prospective Study. J Natl Cancer Inst (2020) 112:516–24. doi: 10.1093/jnci/djz166 PMC7225675 31435679

[7] Wang N Wu S Zhao J Chen M Zeng J Lu G. Bile Acids Increase Intestinal Marker Expression *via* the FXR/SNAI2/miR-1 Axis in the Stomach. Cell Oncol (Dordr) (2021) 44:1119–31. doi: 10.1007/s13402-021-00622-z PMC8516775 34510400

[8] Kullak-Ublick GA Stieger B Meier PJ. Enterohepatic Bile Salt Transporters in Normal Physiology and Liver Disease. Gastroenterology (2004) 126:322–42. doi: 10.1053/j.gastro.2003.06.005 14699511

[9] Jia W Xie G Jia W. Bile Acid-Microbiota Crosstalk in Gastrointestinal Inflammation and Carcinogenesis. Nat Rev Gastroenterol Hepatol (2018) 15:111–28. doi: 10.1038/nrgastro.2017.119 PMC5899973 29018272

[10] Perino A Demagny H Velazquez-Villegas L Schoonjans K. Molecular Physiology of Bile Acid Signaling in Health, Disease, and Aging. Physiol Rev (2021) 101:683–731. doi: 10.1152/physrev.00049.2019 32790577

[11] Cook JW Knnaway KM. Production of Tumours in Mice by Deoxycholic Acid. Nature (1940) 145:627–7. doi: 10.1038/145627a0

[12] Bernstein H Bernstein C Payne CM Dvorak K. Bile Acids as Endogenous Etiologic Agents in Gastrointestinal Cancer. World J Gastroenterol (2009) 15:3329–40. doi: 10.3748/wjg.15.3329 PMC2712893 19610133

[13] Fu T Coulter S Yoshihara E Oh TG Fang S Cayabyab F. FXR Regulates Intestinal Cancer Stem Cell Proliferation. Cell (2019) 176:1098–1112.e18. doi: 10.1016/j.cell.2019.01.036 30794774 PMC6701863

[14] Li T Guo H Li H Jiang Y Zhuang K Lei C. MicroRNA-92a-1-5p Increases CDX2 by Targeting FOXD1 in Bile Acids-Induced Gastric Intestinal Metaplasia. Gut (2019) 68:1751–63. doi: 10.1136/gutjnl-2017-315318 PMC6839796 30635407

[15] Ocvirk S O’Keefe SJD. Dietary Fat, Bile Acid Metabolism and Colorectal Cancer. Semin Cancer Biol (2021) 73:347–55. doi: 10.1016/j.semcancer.2020.10.003 33069873

[16] Tatsugami M Ito M Tanaka S Yoshihara M Matsui H Haruma K. Bile Acid Promotes Intestinal Metaplasia and Gastric Carcinogenesis. Cancer Epidemiol Biomarkers Prev (2012) 21:2101–7. doi: 10.1158/1055-9965.EPI-12-0730 23010643

[17] Staels B Fonseca VA. Bile Acids and Metabolic Regulation: Mechanisms and Clinical Responses to Bile Acid Sequestration. Diabetes Care (2009) 32 Suppl 2:S237–245. doi: 10.2337/dc09-S355 PMC2811459 19875558

[18] Martinot E Sèdes L Baptissart M Lobaccaro J-M Caira F Beaudoin C. Bile Acids and Their Receptors. Mol Aspects Med (2017) 56:2–9. doi: 10.1016/j.mam.2017.01.006 28153453

[19] Li M Cai S-Y Boyer JL. Mechanisms of Bile Acid Mediated Inflammation in the Liver. Mol Aspects Med (2017) 56:45–53. doi: 10.1016/j.mam.2017.06.001 28606651 PMC5662014

[20] Chávez-Talavera O Tailleux A Lefebvre P Staels B. Bile Acid Control of Metabolism and Inflammation in Obesity, Type 2 Diabetes, Dyslipidemia, and Nonalcoholic Fatty Liver Disease. Gastroenterology (2017) 152:1679–1694.e3. doi: 10.1053/j.gastro.2017.01.055 28214524

[21] Albaugh VL Banan B Ajouz H Abumrad NN Flynn CR. Bile Acids and Bariatric Surgery. Mol Aspects Med (2017) 56:75–89. doi: 10.1016/j.mam.2017.04.001 28390813 PMC5603298

[22] Sèdes L Martinot E Baptissart M Baron S Caira F Beaudoin C. Bile Acids and Male Fertility: From Mouse to Human? Mol Aspects Med (2017) 56:101–9. doi: 10.1016/j.mam.2017.05.004 28511935

[23] Bernstein C Holubec H Bhattacharyya AK Nguyen H Payne CM Zaitlin B. Carcinogenicity of Deoxycholate, a Secondary Bile Acid. Arch Toxicol (2011) 85:863–71. doi: 10.1007/s00204-011-0648-7 PMC3149672 21267546

[24] Feng H-Y Chen Y-C. Role of Bile Acids in Carcinogenesis of Pancreatic Cancer: An Old Topic With New Perspective. World J Gastroenterol (2016) 22:7463–77. doi: 10.3748/wjg.v22.i33.7463 PMC5011662 27672269

[25] Di Ciaula A Wang DQ-H Molina-Molina E Lunardi Baccetto R Calamita G Palmieri VO. Bile Acids and Cancer: Direct and Environmental-Dependent Effects. Ann Hepatol (2017) 16 Suppl 1:S87–S105. doi: 10.5604/01.3001.0010.5501 29080344

[26] Kundu S Kumar S Bajaj A. Cross-Talk Between Bile Acids and Gastrointestinal Tract for Progression and Development of Cancer and its Therapeutic Implications. IUBMB Life (2015) 67:514–23. doi: 10.1002/iub.1399 26177921

[27] Shneider BL. Intestinal Bile Acid Transport: Biology, Physiology, and Pathophysiology. J Pediatr Gastroenterol Nutr (2001) 32:407–17. doi: 10.1097/00005176-200104000-00002 11396803

[28] Bernstein C Bernstein H Garewal H Dinning P Jabi R Sampliner RE. A Bile Acid-Induced Apoptosis Assay for Colon Cancer Risk and Associated Quality Control Studies. Cancer Res (1999) 59:2353–7. 10344743

[29] Powolny A Xu J Loo G. Deoxycholate Induces DNA Damage and Apoptosis in Human Colon Epithelial Cells Expressing Either Mutant or Wild-Type P53. Int J Biochem Cell Biol (2001) 33:193–203. doi: 10.1016/s1357-2725(00)00080-7 11240376

[30] Ochsenkühn T Bayerdörffer E Meining A Schinkel M Thiede C Nüssler V. Colonic Mucosal Proliferation Is Related to Serum Deoxycholic Acid Levels. Cancer (1999) 85:1664–9. doi: 10.1002/(SICI)1097-0142(19990415)85:8<1664::AID-CNCR4>3.0.CO;2-O 10223558

[31] Grubben MJ van den Braak CC Essenberg M Olthof M Tangerman A Katan MB. Effect of Resistant Starch on Potential Biomarkers for Colonic Cancer Risk in Patients With Colonic Adenomas: A Controlled Trial. Dig Dis Sci (2001) 46:750–6. doi: 10.1023/a:1010787931002 11330408

[32] Bayerdörffer E Mannes GA Richter WO Ochsenkühn T Wiebecke B Köpcke W. Increased Serum Deoxycholic Acid Levels in Men With Colorectal Adenomas. Gastroenterology (1993) 104:145–51. doi: 10.1016/0016-5085(93)90846-5 8419237

[33] Kasbo J Saleem M Perwaiz S Mignault D Lamireau T Tuchweber B. Biliary, Fecal and Plasma Deoxycholic Acid in Rabbit, Hamster, Guinea Pig, and Rat: Comparative Study and Implication in Colon Cancer. Biol Pharm Bull (2002) 25:1381–4. doi: 10.1248/bpb.25.1381 12392101

[34] Bayerdörffer E Mannes GA Ochsenkühn T Dirschedl P Wiebecke B Paumgartner G. Unconjugated Secondary Bile Acids in the Serum of Patients With Colorectal Adenomas. Gut (1995) 36:268–73. doi: 10.1136/gut.36.2.268 PMC1382415 7883228

[35] Jenkins GJS Harries K Doak SH Wilmes A Griffiths AP Baxter JN. The Bile Acid Deoxycholic Acid (DCA) at Neutral pH /Activates NF-kappaB and Induces IL-8 Expression in Oesophageal Cells In Vitro. Carcinogenesis (2004) 25:317–23. doi: 10.1093/carcin/bgh032 14656946

[36] Jankowski JA Harrison RF Perry I Balkwill F Tselepis C. Barrett’s Metaplasia. Lancet (2000) 356:2079–85. doi: 10.1016/S0140-6736(00)03411-5 11145505

[37] Fein M Fuchs KH Stopper H Diem S Herderich M. Duodenogastric Reflux and Foregut Carcinogenesis: Analysis of Duodenal Juice in a Rodent Model of Cancer. Carcinogenesis (2000) 21:2079–84. doi: 10.1093/carcin/21.11.2079 11062171

[38] Domellöf L Reddy BS Weisburger JH. Microflora and Deconjugation of Bile Acids in Alkaline Reflux After Partial Gastrectomy. Am J Surg (1980) 140:291–5. doi: 10.1016/0002-9610(80)90024-0 7406138

[39] Graffner H Florén CH Nilsson A. Conjugated Bile Salts in Gastric Aspirates After Gastric Resection. Scand J Gastroenterol (1984) 19:116–8. doi: 10.1080/00365521.1984.12005695 6710073

[40] Miwa K Sahara H Segawa M Kinami S Sato T Miyazaki I. Reflux of Duodenal or Gastro-Duodenal Contents Induces Esophageal Carcinoma in Rats. Int J Cancer (1996) 67:269–74. doi: 10.1002/(SICI)1097-0215(19960717)67:2<269::AID-IJC19>3.0.CO;2-6 8760598

[41] Miyashita T Miwa K Fujimura T Ninomiya I Fushida S Shah FA. The Severity of Duodeno-Esophageal Reflux Influences the Development of Different Histological Types of Esophageal Cancer in a Rat Model. Int J Cancer (2013) 132:1496–504. doi: 10.1002/ijc.27824 22961324

[42] Munemoto M Mukaisho K-I Miyashita T Oyama K Haba Y Okamoto K. Roles of the Hexosamine Biosynthetic Pathway and Pentose Phosphate Pathway in Bile Acid-Induced Cancer Development. Cancer Sci (2019) 110:2408–20. doi: 10.1111/cas.14105 PMC6676276 31215094

[43] Li D Zhang J Yao WZ Zhang DL Feng CC He Q. The Relationship Between Gastric Cancer, its Precancerous Lesions and Bile Reflux: A Retrospective Study. J Dig Dis (2020) 21:222–9. doi: 10.1111/1751-2980.12858 PMC7317534 32187838

[44] Barros R Freund J-N David L Almeida R. Gastric Intestinal Metaplasia Revisited: Function and Regulation of CDX2. Trends Mol Med (2012) 18:555–63. doi: 10.1016/j.molmed.2012.07.006 22871898

[45] Zhou H Ni Z Li T Su L Zhang L Liu N. Activation of FXR Promotes Intestinal Metaplasia of Gastric Cells *via* SHP-Dependent Upregulation of the Expression of CDX2. Oncol Lett (2018) 15:7617–24. doi: 10.3892/ol.2018.8342 PMC5962842 29849798

[46] Ni Z Min Y Han C Yuan T Lu W Ashktorab H. TGR5-Hnf4α Axis Contributes to Bile Acid-Induced Gastric Intestinal Metaplasia Markers Expression. Cell Death Discov (2020) 6:56. doi: 10.1038/s41420-020-0290-3 32655894 PMC7338499

[47] Chen C-H Lin C-L Kao C-H. The Effect of Cholecystectomy on the Risk of Colorectal Cancer in Patients With Gallbladder Stones. Cancers (Basel) (2020) 12:E550. doi: 10.3390/cancers12030550 32120781 PMC7139669

[48] Petrera M Paleari L Clavarezza M Puntoni M Caviglia S Briata IM. The ASAMET Trial: A Randomized, Phase II, Double-Blind, Placebo-Controlled, Multicenter, 2 × 2 Factorial Biomarker Study of Tertiary Prevention With Low-Dose Aspirin and Metformin in Stage I-III Colorectal Cancer Patients. BMC Cancer (2018) 18:1210. doi: 10.1186/s12885-018-5126-7 30514262 PMC6280542

[49] Morales DR Morris AD. Metformin in Cancer Treatment and Prevention. Annu Rev Med (2015) 66:17–29. doi: 10.1146/annurev-med-062613-093128 25386929

[50] Vallianou NG Kostantinou A Kougias M Kazazis C. Statins and Cancer. Anticancer Agents Med Chem (2014) 14:706–12. doi: 10.2174/1871520613666131129105035 24295174

[51] Liu T Song X Khan S Li Y Guo Z Li C. The Gut Microbiota at the Intersection of Bile Acids and Intestinal Carcinogenesis: An Old Story, Yet Mesmerizing. Int J Cancer (2020) 146:1780–90. doi: 10.1002/ijc.32563 31291465

