# Supplementary material for: Serum Total Bile Acids in Relation to Gastrointestinal Cancer Risk: A Retrospective Study
Source: Front Oncol. 2022 Jun 8;12:859716. doi: 10.3389/fonc.2022.859716 (PMC9213662; doi:10.3389/fonc.2022.859716)
Supplement: Supplementary file 1 [file Table_1.doc]

**Serum total bile acids in relation to gastrointestinal cancer risk: a retrospective study**

**Contents**

**Table S1.** ROC curve parameters of various diagnostic indexes of GICs.

**Table S1. ROC curve parameters of various diagnostic indexes of GICs.**

|  | AUC | Criterion | Sensitivity (%) | Specificity (%) | Youden index | *P* ^a^ |
| --- | --- | --- | --- | --- | --- | --- |
| EC | | | | | | |
| TBA | 0.640 | >2.6 | 65.7 | 55.4 | 0.210 | <0.001 |
| CEA | 0.547 | >1.70 | 58.8 | 47.3 | 0.091 | <0.001 |
| AFP | 0.521 | ≤2.13 | 34.6 | 74.0 | 0.086 | 0.057 |
| CA199 | 0.514 | >6.05 | 75.3 | 27.6 | 0.029 | 0.189 |
| CA125 | 0.504 | ≤4.11 | 10.4 | 93.1 | 0.035 | 0.724 |
| TBA+CEA | 0.640 | >0.471^b^ | 65.7 | 55.4 | 0.210 | <0.001 |
| GC | | | | | | |
| TBA | 0.521 | >7.94 | 13.0 | 96.0 | 0.090 | 0.067 |
| CEA | 0.601 | >2.82 | 40.1 | 77.4 | 0.175 | <0.001 |
| AFP | 0.512 | ≤2.17 | 33.1 | 72.9 | 0.060 | 0.309 |
| CA199 | 0.554 | >24.66 | 19.9 | 94.5 | 0.144 | <0.001 |
| CA125 | 0.625 | >9.19 | 63.7 | 55.8 | 0.195 | <0.001 |
| CRC | | | | | | |
| TBA | 0.504 | ≤8.00 | 90.9 | 3.8 | 0.054 | 0.713 |
| CEA | 0.721 | >3.46 | 48.5 | 86.9 | 0.354 | <0.001 |
| AFP | 0.509 | ≤2.17 | 31.1 | 72.9 | 0.040 | 0.392 |
| CA199 | 0.630 | >16.59 | 37.6 | 82.7 | 0.203 | <0.001 |
| CA125 | 0.643 | >11.05 | 50.4 | 70.0 | 0.204 | <0.001 |
| GICs | | | | | | |
| TBA | 0.552 | >7.65 | 14.2 | 95.3 | 0.096 | <0.001 |
| CEA | 0.627 | >3.46 | 32.7 | 86.9 | 0.196 | <0.001 |
| AFP | 0.514 | ≤2.17 | 33.2 | 72.9 | 0.061 | 0.115 |
| CA199 | 0.569 | >17.33 | 27.5 | 84.5 | 0.120 | <0.001 |
| CA125 | 0.520 | ≤0 | 94.4 | 9.5 | 0.039 | <0.001 |

**Note**: a: *P* represents comparison with AUC=0.5; b: The criterion of joint diagnosis is the probability value obtained during logistics regression. **Abbreviation**: TBA, total bile acid; EC, esophageal cancer; GC, gastric cancer; CRC, colorectal cancer; GICs, gastrointestinal cancers; OR, odds ratio; CI, confidence interval.
